# Prevalence of loneliness and social isolation amongst individuals with severe mental disorders: a systematic review and meta-analysis

**DOI:** 10.1017/S2045796025000228

**Published:** 2025-04-15

**Authors:** André Hajek, Razak M. Gyasi, Supa Pengpid, Karl Peltzer, Karel Kostev, Pinar Soysal, Lee Smith, Louis Jacob, Nicola Veronese, Hans-Helmut König

**Affiliations:** 1 Department of Health Economics and Health Services Research, University Medical Center Hamburg-Eppendorf, Hamburg Center for Health Economics, Hamburg, Germany; 2 African Population and Health Research Center, Nairobi, Kenya; 3 Faculty of Health, National Centre for Naturopathic Medicine, Southern Cross University, Lismore, NSW, Australia; 4 Department of Health Education and Behavioral Sciences, Faculty of Public Health, Mahidol University, Bangkok, Thailand; 5 Department of Public Health, Sefako Makgatho Health Sciences University, Pretoria, South Africa; 6 Department of Healthcare Administration, College of Medical and Health Science, Asia University, Taichung, Taiwan; 7 Department of Psychology, University of the Free State, Bloemfontein, South Africa; 8 Department of Psychology, College of Medical and Health Science, Asia University, Taichung, Taiwan; 9 University Hospital Marburg, Philipps-University Marburg, Baldingerstraße, Marburg, Germany; 10 Department of Geriatric Medicine, Faculty of Medicine, Bezmialem Vakif University, Istanbul, Turkiye; 11 Centre for Health, Performance and Wellbeing, Anglia Ruskin University, Cambridge, UK; 12 Department of Physical Medicine and Rehabilitation, AP-HP, Lariboisière-Fernand Widal Hospital, Université Paris Cité, Paris, France; 13 Université Paris Cité, INSERM U1153, CRESS, Epidemiology of Ageing and Neurodegenerative Diseases (EpiAgeing), Paris, France; 14 Research and Development Unit, Parc Sanitari Sant Joan de Déu, CIBERSAM, ISCIII, Dr. Antoni Pujadas, 42, Sant Boi de Llobregat, Barcelona, Spain; 15 Department of Medicine, Geriatric Unit, University of Palermo, Palermo, Italy

**Keywords:** bipolar disorder, loneliness, schizophrenia, serious mental illness, severe mental disorder, social isolation

## Abstract

**Aims:**

A systematic review and meta-analysis was conducted to investigate the prevalence and antecedents/outcomes of loneliness and social isolation among individuals with severe mental disorders (SMD), such as schizophrenia, schizoaffective disorder, bipolar disorder or major depressive disorder.

**Methods:**

Five well-known electronic databases (PubMed, PsycINFO, CINAHL, Web of Science and Scopus) were searched (plus a hand search). Observational studies that report the prevalence and, if available, antecedents and consequences of loneliness/isolation among individuals with SMD were included. Key characteristics were extracted, and a meta-analysis was performed. Our systematic review was preregistered on PROSPERO (ID: CRD42024559043). The PRISMA guidelines were followed. The Joanna Briggs Institute (JBI) standardized critical appraisal tool developed for prevalence studies was applied to assess the quality of the included studies.

**Results:**

The initial search yielded 4506 records, and after duplicate removal and screening, a total of 10 studies were finally included. The studies included used data from Europe, Asia, North America, and Oceania. Two studies employed a longitudinal design, while all other studies had a cross-sectional design. Most of the studies included between 100 and 500 individuals with SMD. All studies involved both male and female participants, with women typically comprising about 40% of the sample. The average age of participants often ranged from approximately 30 to 40 years. The estimated prevalence of loneliness was 59.1% (95% CI: 39.6% to 78.6%, *I*^2^ = 99.3, *P* < .001) among individuals with any diagnosis of SMD. Furthermore, the estimated prevalence of objective social isolation was 63.0% (95% CI: 58.6% to 67.4%) among individuals with schizophrenia or schizophrenia spectrum disorder. The quality of the studies was moderate to good. Subjective well-being and depressive symptoms in particular were found to contribute to loneliness in the included studies.

**Conclusions:**

The present systematic review with meta-analysis identified high levels of loneliness and objective social isolation among those with SMD. These findings stress the importance of monitoring and addressing social needs in this vulnerable group, which may have a positive effect on the life quality of individuals with SMD. Future research in neglected regions (e.g. South America and Africa) is recommended. Different diagnoses within severe mental disorders should be distinguished in future studies. Furthermore, additional longitudinal studies are required to explore the antecedents and consequences of loneliness and social isolation among individuals with SMD.

## Introduction

Severe mental disorders (SMD, also known as serious mental illness) includes illnesses such as schizophrenia, schizoaffective disorder, bipolar disorder or major depressive disorder (De Hert *et al.*, 2011). They are characterized by serious functional impairment and limiting one or more major activities of life. Individuals with SMD may also spend significant time in institutionalized surroundings (Valdes-Stauber and Kilian, 2015).

High prevalence rates of SMD have been reported in different populations such as conflict-affected populations (Charlson *et al.*, 2019) or individuals with substance use disorders (Mmbapin *et al.*, 2019). Additionally, a previous meta-analysis showed that physical disorders such as hypertension or tardive dyskinesia are common among individuals with SMD (Mmbapin *et al.*, 2019). Individuals with SMD also report markedly lower quality of life compared to healthy populations (Evans *et al.*, 2007). Moreover, there is a high economic burden associated with SMD (Christensen *et al.*, 2022; Hakulinen *et al.*, 2020). Individuals with SMD also have a shorter life expectancy, e.g. due to comorbidities or suicidality (Schneider *et al.*, 2019).

Moreover, those with SMD can experience other negative psychosocial outcomes including loneliness (i.e. negative emotion where they feel a discrepancy between desired and actual social relationships (Perlman and Peplau, 1981)) and objective (i.e. a lack of social activities (Hajek and König, 2021)) and perceived social isolation (feeling of not belonging to the society). For example, those with SMD may withdraw from social relationships owing to reported less consummatory social pleasure compared to controls (Abel *et al.*, 2023). Other possible reasons are that individuals with SMD may find it difficult to initiate or maintain conversations or participate in social activities due to lack of motivation, persistent mood swings or hallucinations. Moreover, individuals with SMD may feel stigmatized, judged or misunderstood, leading them to withdraw from social activities (Ertugrul and Uluğ, 2004). Individuals with SMD may have strained relationships with relatives and friends due to SMD (Stevens *et al.*, 2009). The relationships could be perceived as rather unidirectional, as help is often needed. Additionally, family caregivers of individuals with SMD also frequently report a high burden (Cham *et al.*, 2022; Yükü and Derleme, 2017). Moreover, when patients are subject to guardianship, they have a higher likelihood of being institutionalized (Valdes-Stauber and Kilian, 2015). Such potentially challenging social support networks may increase loneliness and social isolation. SMD may also be accompanied by physical limitations (Nuoffer *et al.*, 2022), which can make it difficult to participate in social activities and events. This can also increase feelings of loneliness and social isolation. Indeed, a previous meta-analysis showed a moderate association between loneliness and psychosis [*r* = .32, 95% confidence interval (CI): .20 to .44] (Michalska da Rocha *et al.*, 2017). Other research showed higher loneliness levels among individuals with schizophrenia compared to non-psychiatric controls (Eglit *et al.*, 2018).

Previous research has reported the average level of loneliness among individuals with SMD (Fortuna *et al.*, 2021). There are also some studies reporting the prevalence of loneliness or objective social isolation among individuals with SMD (Badcock *et al.*, 2015; Machetanz *et al.*, 2023; Suman *et al.*, 2023). Previous work has also focused, for example, on loneliness in specific SMDs, such as psychosis, based on a systematic review (Lim *et al.*, 2018) or on the meta-analytic association between loneliness and psychosis (Michalska da Rocha *et al.*, 2017). However, there is a lack of a systematic review and meta-analysis summarizing the present evidence (in terms of prevalence rates of loneliness and isolation among individuals with SMD). Therefore, our aim was to determine the prevalence of loneliness and social isolation among individuals with SMD; and to further explore the antecedents and consequences of loneliness/isolation in this vulnerable group.

In addition to hereditary factors and biochemical imbalances, environmental influences can contribute to SMD (Ghallab and Elassal, 2024; Schmitt *et al.*, 2014; Schwab and Wildenauer, 2013). Environmental factors such as wars and waves of refugees, which could favour trauma, chronic stress and abuse, could also be relevant (de Silva *et al.*, 2021; Minervini *et al.*, 2023). Against the backdrop of increasing conflict across the world, and an increasing population of asylum seekers, the number of individuals with SMD may increase. This underscores the importance of this current work.

Beyond this, this work may identify antecedents and consequences of loneliness/social isolation among individuals with SMD. Furthermore, by summarizing the existing evidence, we may identify current knowledge gaps which could inspire future studies. Performing a meta-analysis can also provide much more accurate results than single studies, providing needed evidence for intervention and political action. We also consider possible subgroups of studies. This can provide valuable information about which factors are important for the prevalence of loneliness and isolation among individuals with SMD.

## Materials and methods

The present study followed The Preferred Reporting Items for Systematic Reviews and Meta-Analysis (PRISMA) guidelines (Page *et al.*, 2021) and followed a pre-registered protocol (International Prospective Register of Systematic Reviews, PROSPERO CRD42024559043). We did not make any amendments to this. Importantly, we initially planned to conduct an additional meta-regression. However, owing to a small number of studies this was not performed (Higgins *et al.*, 2024).

In July 2024, an electronic search was performed across five databases: PubMed, Psychological Information Database (PsycINFO), Cumulative Index to Nursing and Allied Health Literature (CINAHL), Web of Science and Scopus. Our search strategy and choice of databases were based on advice from a librarian (please see Supplement 1 regarding the search strategy).

Two reviewers (A.H., H.H.K.) independently conducted study selection in two steps (1: title/abstract screening; 2: full-text screening). We also reviewed the references of all studies that met the final inclusion criteria and examined the studies that cited the included studies (manual search). Conflicting opinions on the inclusion of studies were resolved through discussion.

The following inclusion criteria were applied: (i) Cross-sectional and longitudinal observational studies investigating the prevalence of loneliness or social isolation among individuals with SMD, (ii) usage of appropriate tools for assessing key variables, (iii) studies available in English or German and published in peer-reviewed scientific journals. Studies exclusively examining children or adolescents were excluded. Moreover, studies reporting only the mean, but not the prevalence, of loneliness or social isolation among individuals with SMD were excluded. An appropriate assessment for loneliness/isolation mainly followed the COnsensus-based Standards for the selection of health Measurement INstruments (COSMIN) guidelines (Prinsen *et al.*, 2018). Documented diagnosis following the 10^th^ Revision of the International Classification of Diseases (ICD-10) or Diagnostic and Statistical Manual of Mental Disorders (DSM) criteria was mainly applied for SMD.

We did not investigate grey literature. This decision was made to ensure a minimum level of quality. There were no restrictions as to the place and time of the studies. A pre-test of 100 titles/abstracts was carried out before the final inclusion criteria were determined. However, our inclusion criteria did not change. One author (A.H.) conducted the data extraction, and a second author (H.H.K.) carefully checked the data extraction. Data extraction included study design, measure of loneliness/isolation, assessment of SMD, sample characteristics, analytical approach and main findings. If data were incomplete or unclear, the authors of the studies were contacted.

The Joanna Briggs Institute (JBI) standardized critical appraisal tool developed for prevalence studies was applied to evaluate study quality (Moola *et al.*, 2017). The final total score varies from 0 to 9, with higher scores reflecting better study quality and lower risk of bias. One reviewer (A.H.) carried out the assessment, which was checked by a second reviewer (H.H.K.).

For the meta-analysis, a random effects model was used as heterogeneity between studies can be assumed. In accordance with current recommendations, the *I*^2^ statistic was used to assess heterogeneity between studies (with *I*^2^ values of 25–50% were classified as low, 50–75% as moderate and 75% or more as high heterogeneity (Higgins *et al.*, 2003)).

Of note that there are differences between specific SMDs (e.g. between major depressive disorder and schizophrenia). However, individuals with different SMD share many similarities in terms of emotional and social challenges (e.g. tendencies to social withdrawal, negative self-perception and impairments in communication), which may contribute to the quality of personal relationships. Thus, we argue that a meta-analysis focusing on the prevalence of loneliness and social isolation among the group of individuals with SMD is meaningful. However, we also conducted meta-analyses for individuals with different SMD.

In this current work, the severity of loneliness and social isolation – such as moderate or severe loneliness – was not considered due to a lack of available data. The presence of loneliness was defined as roughly comparable across the included studies. However, some differences are worth noting. For example, while Fortuna *et al.* (2024) used the established cut-off of 6 or higher for the University of California, Los Angeles Loneliness Scale (3-item version; UCLA-3), Heron *et al*. used a cut-off of 7 or higher for the UCLA-3 (Heron *et al.*, 2022). Moreover, Suman *et al.* (2023) also used the UCLA-3 and indicated loneliness if individuals responded with at least ‘sometimes/often’ (2) to any of the items. The recommended cut-off for the De Jong Gierveld (DJG) tool was applied in the study by Dell *et al.* (2019). However, this cut-off can result in very high prevalence rates (Hajek and König, 2022). Thus, an alternative cut-off also exists, which produces more conservative prevalence rates of loneliness (Hansen and Slagsvold, 2016). Of note, Dell *et al.* (2019) solely used the recommended cut-off. For social isolation, established cut-offs were used (see Table 1 for additional details). This should be kept in mind when interpreting the results of the meta-analyses.

**Table 1.** Study overview and key findings

Author (Year)	Country	Assessment of loneliness/social isolation; SMD	Sample and study type	Time of data collection	Sample size, age in total sample and sex ratio	Results: prevalence of loneliness/isolation (%)	Results: prevalence stratified by sex	Results: antecedents/consequences
Badcock *et al.* (2015)	Australia	Loneliness: Single item	Second Australian National Survey of Psychosis	2010	*N* = 1603 with psychotic disorders	Loneliness (among individuals with (1) schizophrenia, (2) schizoaffective disorder, (3) bipolar disorder with psychotic features, (4) depressive psychosis, (5) delusional disorders and other non-organic psychosis): 634/835 (75.9%), 240/287 (83.6%), 267/314 (85.0%), 75/80 (93.8%), 65/87 (74.7%)	Not reported	Loneliness: significantly associated with loss of pleasure (OR = 2.0, 95% CI: 1.2 to 3.3, *P* < .05) and subjective thought disorder (OR: 1.4, 95% CI: 1.0 to 2.0, *P* < .05)
		Psychotic disorders: In accordance with the ICD-10 criteria			Mean age (SD): 38.2 (11.0)			
					Female: 38.6%			
Dell *et al.* (2019)	USA	Loneliness: DJG-tool	Individuals receiving Community Psychiatric Rehabilitation Program services	June 2017 to December 2017	*N* = 95 individuals with severe mental illness	Loneliness: 67/95 (70.5%)	Not reported	Emotional loneliness: significantly associated with depressive symptoms (β = .74, *P* < .001)
		SMD: According to Substance Abuse and Mental Health Services Administration			Mean age (SD): 59.4 (SD: 6.5)			
					Female: 45.3%			
Fortuna *et al.* (2024)	USA	Loneliness: UCLA-3	New York City boroughs	April 2023	*N* = 519 individuals with SMD (schizophrenia spectrum disorder, bipolar disorder and major depressive disorder)	Loneliness among individuals with schizophrenia spectrum disorder: 81/314 (25.8%)	Not reported	High loneliness among women: Significantly associated with being Hispanic (vs. White, OR: .079, 95% CI: .006 to .971, *P* = .047) and residing in scattered site housing (vs. congregate, OR: 2.307, 95% CI: 1.131 to 4.707, *P* = .022)
		SMD: Primary ICD diagnosis data (F2x and F3x) were used based on electronic health records			Mean age (SD): 63.2 (6.2)	Loneliness among individuals with bipolar disorder: 23/79 (29.1%)		
					Female: 38.5%	Loneliness among individuals with major depressive disorder: 33/126 (26.2%)		
Hamaideh (2021)	Jordan	Loneliness: Revised UCLA Loneliness Scale	Data from National Center for Mental Health	Not reported	*N* = 230 patients with schizophrenia	Loneliness (moderate/high to high level): 160/230 (69.6%) SM	Not reported	Higher loneliness: significantly associated with lower satisfaction with life (β = −.40, *P* < .001), low social support from friends (β = −.36, *P* < .001), higher duration of treatment (β = .15, *P* < .001)
		Schizophrenia: Diagnosis of Schizophrenia			Mean age (SD): 36.7 (10.4)			
					Female: 50.9%			
Heron *et al.* (2022)	United Kingdom	Loneliness: UCLA-3	Subsection of the “Closing the Gap” clinical cohort	July 2020 to December 2020	*N* = 358 individuals with severe mental illness	Loneliness (UCLA − 3): 125/358 (34.9%)	Not reported	Loneliness: significantly associated with living alone (vs. living alone, adjusted OR = 2.04, 95% CI 1.21 − 3.43, *P* = .01), living in an area with high index of multiple deprivation (vs. area with very low index, adjusted OR = 2.49, 95% CI 1.04 − 5.95, *P* = .04), and younger age (adjusted OR = − .98, 95% CI .964−.997, *P* = .02)
		SMD: Documented diagnoses of bipolar disorder (ICD F31.X or DSM-equivalent), or schizophrenia or delusional/psychotic illness (ICD-10 F20.X & F22.X or DSM equivalent)			Mean age (SD): 50.5 (SD not reported)	Loneliness “often” (Single-item): 107/358 (29.9%)		
					Female: 47.4%	Loneliness “some of the time” (Single-item): 129/358 (36.0%)		
Machetanz *et al.* (2023)	Switzerland	Objective social isolation: based on three conditions (regarding social factors)	Court-mandated inpatient treatment	1982 to 2016	*N* = 320 forensic psychiatric patients with a schizophrenia spectrum disorder	Objective social isolation: 226/320 (70.6%)	Objective social isolation (among women): 21/29 (72.4%)	Differentiate between patients with and without objective social isolation: attention disorder, alogia, crime motivated by ego disturbances, PANSS score, and a history of negative symptoms
		Schizophrenia spectrum disorder: According to ICD-10 (F2x)			Mean age (SD): 34.3 (10.4)		Objective social isolation (among men): 205/291 (70.4%)	
					Female: 9.1%			
Okruszek *et al.* (2023)	Poland	Objective social isolation: LSNS − 6	A total of 93 adult patients with schizophrenia were enrolled in the study	Not reported	*N* = 93 individuals with schizophrenia	Objective social isolation: 32/93 (34.4%)	Not reported	Higher loneliness: associated with social threat bias
		Schizophrenia: According to the ICD − 10 criteria			Mean age (SD): 33.1 (7.8)			
					Female: 36.6%			
Stain *et al.* (2012)	Australia	Equal to Badcock *et al.*, 2015	“Second Australian National Survey of Psychosis”	2010	*N* = 1780 with psychotic disorders	Loneliness: 1426/1780 (80.1%)	Loneliness among women: 601/725 (82.9%)	Not reported
					Mean age (SD): 38.4 (11.2)		Loneliness among men: 825/1055 (78.2%)	
					Female: 40.4%			
Suman *et al.* (2023)	India	Loneliness: UCLA-3	Patients of schizophrenia in clinical remission	October 2019 to March 2020	*N* = 160 individuals with schizophrenia	Loneliness among individuals with schizophrenia: 128/160 (80%)	Not reported	Loneliness: largely explained by quality of life (29%), followed by hopelessness (8%) and discrimination (2%).
		Schizophrenia: Diagnosed according to DSM-5 criteria			Mean age (SD): 35.0 (9.1)			
					Female: 40.6%			
Valeri *et al.* (2023)	USA	Loneliness: Single-item	Part of the “Intensive Longitudinal Health Behavior Network project”	2015 and 2021 (and followed for up to four years)	*N* = 7 with SMD (with 634 daily observations over a four-month period)	“Moderate or extreme” loneliness (2 months before shelter-in-place order): 182/348 (52.3%)	Not reported	Increases in loneliness: not significantly associated with shelter-in-place orders (OR = 2.10, *P* = .08)
		SMD: Psychosis			Mean age: (SD): 29 (SD not reported)			
					Female: 57.1%			

*Legend*: CI: Confidence Interval; DJG-tool: De Jong Gierveld loneliness tool; DSM: Diagnostic and Statistical Manual of Mental Disorders; ICD-10: 10th Revision of the International Classification of Diseases; LSNS-6: Lubben Social Network Scale (6-item version); OR: Odds Ratio; PANSS: Positive and Negative Syndrome Scale; SD: Standard Deviation; SMD: Severe Mental Disorder; UCLA-3: University of California, Los Angeles Loneliness Scale (3-item version).

Our intention was to conduct a funnel plot and perform the Egger test to identify a potential publication bias (Egger *et al.*, 1997). Since the number of studies was small, it was not performed. Stata 18.0 (College Station, Texas, USA) was used for meta-analysis. The ‘metaprop’ tool was used (Nyaga *et al.*, 2014).

## Results

### Study overview

The search process is displayed in Supplement 2 (Page *et al.*, 2021). In total, 4506 records were initially identified. After removing duplicates, 3616 entries remained for screening. Following this phase, 42 full-text articles were assessed in the subsequent step. Most of these studies did not fulfil the inclusion criteria, often due to their failure to present prevalence data (e.g. average loneliness scores were, in fact, often reported) for individuals with SMD. In this current work, 10 studies (Badcock *et al.*, 2015; Dell *et al.*, 2019; Fortuna *et al.*, 2024; Hamaideh, 2021; Heron *et al.*, 2022; Machetanz *et al.*, 2023; Okruszek *et al.*, 2023; Stain *et al.*, 2012; Suman *et al.*, 2023; Valeri *et al.*, 2023) were finally included. One of these (Hamaideh, 2021) was identified in the hand reference search.

Table 1 summarizes the main characteristics and key findings of the studies included in this systematic review and meta-analysis (more details: Supplement 3). In all cases, findings of adjusted models are presented (last column).

The date of publication varied from 2012 to 2023. Seven studies were published in 2021 or later. The studies included used data from Europe (three studies, one study each from the UK, Poland and Switzerland), Asia (two studies, one study each from Jordan and India), North America (three studies from the USA) and Oceania (two studies from Australia). Two studies employed a longitudinal design (Machetanz *et al.*, 2023; Valeri *et al.*, 2023), while the other studies had a cross-sectional design. Some of the studies relied on well-established samples such as the ‘Second Australian National Survey of Psychosis’; other studies used data from smaller and more selective hospital-based samples.

The majority of the studies included approximately 100 to 500 individuals with SMD. All studies included both male and female participants, with the proportion of women often being around 40% (and frequently falling between 35% and 50%). The average age of the participants typically ranged from around 30 to 40 years. Three studies had an average age of 50 years and over. Some studies used the UCLA tool (Hughes *et al.*, 2004), the DJG tool (Gierveld and Tilburg, 2006; De Jong-Gierveld and Kamphuls, 1985) and single-item tools. To quantify objective social isolation, one study employed the LSNS-6 tool (Lubben *et al.*, 2006) (with an established cut-off), and one study used a modified version (Steptoe *et al.*, 2013) of the Berkman–Syme Social Network Index (Berkman and Syme, 1979). Perceived social isolation was not investigated in any studies. SMD assessment was mainly based on documented diagnosis following ICD-10 criteria.

Eight studies reported the prevalence of loneliness among individuals with SMD (Badcock *et al.*, 2015; Dell *et al.*, 2019; Fortuna *et al.*, 2024; Hamaideh, 2021; Heron *et al.*, 2022; Stain *et al.*, 2012; Suman *et al.*, 2023; Valeri *et al.*, 2023). Of these, two studies (Badcock *et al.*, 2015; Fortuna *et al.*, 2024) reported diagnosis-stratified prevalence rates, and one study (Stain *et al.*, 2012) reported sex-stratified prevalence rates. In addition, two studies (Machetanz *et al.*, 2023; Okruszek *et al.*, 2023) reported the prevalence of objective social isolation among individuals with SMD. Of these, one study (Machetanz *et al.*, 2023) reported sex-stratified prevalence rates. Of note, none of the studies reported both the prevalence of both loneliness and social isolation.

### Meta-analysis

**Figure 1.**
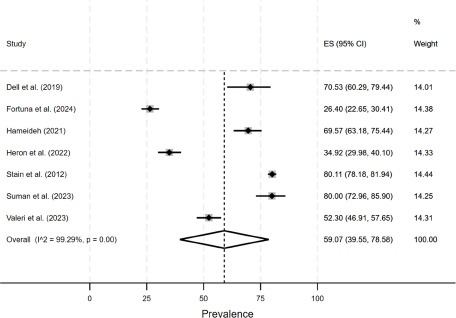
Meta-analysis (loneliness among individuals with any diagnosis of SMD).

The estimated prevalence of loneliness was 59.1% (95% CI: 39.6% to 78.6%, *I*^2^ = 99.3, *P* < .001) among individuals with any diagnosis of SMD (see Fig. 1). The estimated prevalence of loneliness for groups of interest is displayed in Table 2. Based on single-item tools, the estimated prevalence of loneliness was 77.0% (95% CI: 75.3% to 78.8%) among individuals with any diagnosis of SMD, whereas it was 56.2% (95% CI: 33.8% to 78.5%, *I*^2^ = 98.8, *P* < .01) in this group when multi-item tools were used. Stratified by diagnosis, there was, for example, a high prevalence of loneliness (77.5%, 95% CI: 73.9% to 81.2%) among individuals with bipolar disorder. Sex-stratified meta-analyses were not possible due to a small number of studies.

**Table 2.** Subgroup analysis of the pooled prevalence of loneliness

Characteristics	Subgroups	Number of studies	Prevalence	95% CI	*I*^2^ (%), *P*-value
Proportion of female individuals	Lower than 50%	5	58.3	31.7 to 85.0	99.5, *P* < .01
	At least 50%	2	59.9	55.9 to 63.8	
Mean age	Lower than 40 years	4	70.6	57.5 to 83.6	97.1, *P* < .01
	At least 40 years	3	43.4	24.0 to 62.9	97.4, *P* < .01
Time of data collection	Before the Covid-19 pandemic	3	78.5	74.2 to 82.8	50.5, *P* = .13
	During the Covid-19 pandemic	2	43.1	39.5 to 46.7	
Region	Asia	2	74.6	70.3 to 78.9	
	North America	3	49.5	25.2 to 73.7	98.2, *P* < .01
Tools used to quantify loneliness	Single-item	2	77.0	75.3 to 78.8	
	Multi-item tools	5	56.2	33.8 to 78.5	98.8, *P* < .01
Quality assessment score	Score of at least 8	2	74.5	72.8 to 76.3	
	Score of 7 or less	5	59.6	37.9 to 81.4	98.7, *P* < .01
Diagnosis	Schizophrenia	3	75.2	70.3 to 80.1	66.3, *P* = .05
	Schizophrenia (including Schizophrenia Spectrum Disorder)	4	62.8	38.0 to 87.6	99.1, *P* < .01
	Bipolar disorder	2	77.5	73.9 to 81.2	
	SMD (explicitly referring to this term and without further differentiation)	5	52.8	26.8 to 78.8	99.5, *P* < .01

**Figure 2.**
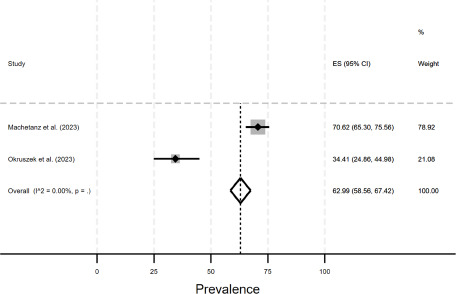
Meta-analysis (social isolation among individuals with schizophrenia or schizophrenia spectrum disorder).

The estimated prevalence of objective social isolation equaled 63.0% (95% CI: 58.6% to 67.4%) among individuals with schizophrenia or schizophrenia spectrum disorder (see Fig. 2). Further meta-analyses (e.g. stratified by sex) were not possible due to a lack of studies.

### Loneliness: antecedents and outcomes

In the two studies that treated loneliness as an antecedent, poor satisfaction with life (Hamaideh, 2021) or lower quality of life (Suman *et al.*, 2023) significantly contributed to loneliness. There was mixed evidence regarding the association between living arrangements and loneliness (Fortuna *et al.*, 2024; Heron *et al.*, 2022; Valeri *et al.*, 2023). Other antecedents were mainly considered in single studies. A few studies showed that certain depressive symptoms, such as hopelessness (Suman *et al.*, 2023) or anhedonia (Badcock *et al.*, 2015), significantly contributed to loneliness. One study showed that emotional, but not social loneliness significantly contributed to depressive symptoms (Dell *et al.*, 2019).

### Social isolation: antecedents and outcomes

Only one study (Machetanz *et al.*, 2023) explicitly examined objective social isolation among patients with schizophrenia spectrum disorders (forensic psychiatric patients; inpatient). Based on a machine learning model, this study showed that attention disorder, alogia, crime motivated by ego disturbances, Positive and Negative Syndrome Scale (PANSS) (Kay *et al.*, 1987) score, and a history of negative symptoms could differentiate between patients with and without social isolation with a balanced accuracy of 69% (area under the curve: .74). Other studies did not examine the antecedents and outcomes of social isolation among individuals with SMD.

### Quality assessment/risk of bias assessment

Quality assessment/risk of bias assessment is depicted in Supplement 4. The average score was 6.4 [standard deviation (SD) = 1.3; 5 to 8], reflecting a moderate to good overall level, with some risk of bias. The most common shortcoming was that the response rate was not explicitly stated.

## Discussion

This systematic review and meta-analysis aimed to determine the prevalence and antecedents/consequences of loneliness/social isolation among individuals with SMD. Our key findings were as follows: High prevalence of loneliness was observed among individuals with SMD (also in several subgroups). Moreover, a high prevalence of objective social isolation among individuals with schizophrenia or schizophrenia spectrum disorder was identified. This is the very first systematic review and meta-analysis summarizing studies identifying the prevalence of loneliness/social isolation in this vulnerable group of individuals with SMD.

The identified prevalence rates for loneliness and objective social isolation are approximately twice as high compared to a meta-analysis focusing on individuals aged 65 years and over during the Covid-19 pandemic (Su *et al.*, 2023) (loneliness: 28.6%; social isolation: 31.2%). A further meta-analysis found a prevalence of 27.1% (severe loneliness) and 32.1% (moderate loneliness) for individuals aged 80 years and over (Hajek *et al.*, 2023). Another recent meta-analysis focusing on loneliness/social isolation among the vulnerable group of individuals with mild cognitive impairment (MCI)/dementia also found somewhat lower prevalence rates of around 40% (Hajek and König, 2025). In this respect, it can be assumed that SMD can largely explain such differences in prevalence rates compared to older adults and individuals with MCI/dementia. Those with SMD may frequently struggle to initiate or maintain conversations and engage in social activities due to motivation issues, mood fluctuations, or hallucinations. They may also withdraw from social interactions due to feelings of being judged or misunderstood, coupled with strained relationships that weaken their support network, contributing to increased loneliness and isolation (Ertugrul and Uluğ, 2004). Physical limitations associated with SMD can further impede participation in social events, exacerbating feelings of loneliness or isolation. This may also explain why loneliness prevalence is high – and comparable to individuals aged 80 years and over who also frequently deal with functional impairment (Hajek *et al.*, 2024). Furthermore, it is worth repeating (see methods section) that there is some variety in the present work in relation to the tools used and the cut-offs applied to quantify loneliness.

The meta-analyses for the examined subgroups suggest some potential differences (e.g. lower loneliness scores in older people or in North America compared to Asia), but the number of studies included was quite small in each case. Possible reasons may relate to, among other things, differences in access to health care, stigma and differences in community support systems. More precisely, cultural stigmatization of SMD may be more pronounced in these Asian countries (Jordan and India) compared to North America (Krendl and Pescosolido, 2020). Furthermore, the social networks and support systems for individuals with SMD in such countries are often less developed, which means that assistance available, and the integration of these individuals into society, can be restricted. Additionally, access to professional mental health care and therapy can be limited, making it more difficult to cope with such disorders and leading to feelings of loneliness and isolation.

Due to the small number of studies, these subgroup analyses should be interpreted with great caution. More robust conclusions require significantly more studies. In this context, it would be of interest to include more developed countries from Asia (e.g. South Korea or Japan). Meta-regressions could then also be used in a meaningful way.

A few included studies showed that life satisfaction or quality of life, as well as certain depressive symptoms, can contribute to loneliness. Similar associations have been identified by a previous meta-analysis focusing on the oldest old (Hajek *et al.*, 2023) and other reviews (Ejiri *et al.*, 2021; Wen *et al.*, 2023). This also aligns with previous research showing that the overall pattern and strengths of correlates are similar between individuals with schizophrenia and non-psychiatric controls (Eglit *et al.*, 2018). However, the limited studies available on the antecedents and consequences of loneliness and isolation in individuals with SMD prevents us from drawing any definitive conclusions. This scarcity of studies highlights the necessity for more in-depth investigation in this area.

The average study quality was moderate to good. A key shortcoming was that nearly all studies did not report the response rate. Since individuals with SMD were surveyed, it can be assumed that unreported response rates in the studies are probably quite low in reality. Assuming that among individuals with SMD, those with even more severe mental health impairments may have a higher likelihood of non-response, sample selection bias may be present in the included studies. As a result, the representativeness of the included studies could be called into question to a certain extent – which should be taken into consideration as a potential shortcoming of the included studies. Moreover, the aforementioned group of individuals with even more severe mental health impairments are expected to have very high loneliness and isolation levels. If such individuals are less likely to participate, then the true prevalence may be underestimated. In addition, some studies were based on small samples and the generalisability was also somewhat limited in some cases due to the recruitment of the individuals.

Several gaps in knowledge were identified. First, studies focusing on the prevalence of social isolation (both, objective and perceived) among individuals with SMD are urgently needed. Although loneliness can be effectively assessed using single-item measures, there is a significant advantage to using multi-item assessments to more fully capture the nuances of loneliness, i.e. different types of loneliness, such as emotional and social loneliness. More uniform cut-offs (or the presentation of prevalence rates based on different cut-offs) would also be strongly desirable in future research. This would significantly improve the comparability of the results. Future studies should also consider various diagnoses within SMD (e.g. distinguishing between schizophrenia, bipolar disorder, or major depressive disorder). This can presumably also have an effect on the *I*^2^. More longitudinal studies are needed to explore the antecedents and consequences of loneliness and social isolation among individuals with SMD. Furthermore, one may assume that individuals with SMD are particularly susceptible to chronic experiences of loneliness and social isolation. Thus, we recommend future research exploring chronic loneliness and isolation in this group in view of the potentially severe consequences (Hajek *et al.*, 2025). Study data were drawn from North America, Europe, Asia and Oceania. However, more geographical diversity is clearly required in future research. We would encourage studies from South America and Africa in particular. Research is also needed as the world now emerges from the Covid-19 pandemic.

Previous research listed some clinical approaches to addressing loneliness, varying from strengthening social abilities, improving social support, as well as discovering ways for social activities to addressing maladaptive social cognition (Perissinotto *et al.*, 2019). For example, cognitive behavioural therapy may help in reframing the harmful beliefs that can affect social interactions (Perissinotto *et al.*, 2019). However, this largely depends on individual needs, which can vary greatly with regard to different life courses and mental illness histories. Lim *et al.* (2018) also summarized that, among other things, strength-based interventions may be beneficial in reducing loneliness in vulnerable groups.

Some strengths and limitations of this work are worth noting. This is the very first systematic review and meta-analysis determining the prevalence of loneliness and social isolation amongst individuals with SMD. Two reviewers carried out relevant steps. Furthermore, our work satisfied the PRISMA guidelines and was preregistered. A potential shortcoming is restricting our search to peer-reviewed articles, which may lead to the exclusion of potentially important studies. However, this approach is in line with the need to include high quality studies. Five large databases were used, although this choice may still have resulted in the exclusion of appropriate studies. However, we believe that searching these large databases (also endorsed by a subject librarian), in combination with the additional hand search, allowed us to identify the great majority of appropriate studies.

In conclusion, for individuals with SMD, objective social isolation and, in particular, loneliness, are major challenges. Such knowledge underscores the importance of monitoring and addressing social needs in this vulnerable group. This knowledge also has the potential to have a positive impact on the quality of life of individuals with SMD. Future research in neglected regions is now required.

## Supporting information

Hajek et al. supplementary material Hajek et al. supplementary material

## Data Availability

All necessary information is provided in the tables and text. The corresponding author can be contacted (a.hajek@uke.de) for further details.

## Supplementary material

The supplementary material for this article can be found at https://doi.org/10.1017/S2045796025000228.
