# Supplementary material for: Prevalence of loneliness and social isolation amongst individuals with severe mental disorders: a systematic review and meta-analysis
Source: Epidemiol Psychiatr Sci. 2025 Apr 15;34:e25. doi: 10.1017/S2045796025000228 (PMC12037343; doi:10.1017/S2045796025000228)
Supplement: Hajek et al. supplementary material [file S2045796025000228sup001.docx]

Supplement to: Prevalence of loneliness and social isolation amongst individuals with severe mental disorders: A systematic review and meta-analysis

Authors: André Hajek*, Razak M. Gyasi, Supa Pengpid, Karl Peltzer, Karel Kostev, Pinar Soysal, Lee Smith, Louis Jacob, Nicola Veronese and Hans-Helmut König

Table of contents

[Supplement 1. Search strategy (PubMed, CINAHL, PsycInfo, Web of Science and Scopus) 2](#_Toc193882954)

[Supplement 2. Flowchart 4](#_Toc193882955)

[Supplement 3. Additional details regarding the included studies 5](#_Toc193882956)

[Supplement 4. Quality assessment/risk of bias assessment. 8](#_Toc193882957)

[References 9](#_Toc193882958)

# Supplement 1. Search strategy (PubMed, CINAHL, PsycInfo, Web of Science and Scopus)

| Database | Search strategy |
| --- | --- |
| PubMed | ("social isolation"[MeSH Terms] OR "social exclu*"[Title/Abstract] OR "social isolat*"[Title/Abstract] OR "lonel*"[Title/Abstract]) AND ("bipolar and related disorders"[MeSH Terms] OR ("depressive disorder, major"[MeSH Terms] OR "depressive disorder"[MeSH Terms]) OR "schizophrenia spectrum and other psychotic disorders"[MeSH Terms] OR "severe mental disorder*"[Title/Abstract] OR "serious mental illness"[Title/Abstract] OR "severe mental illness"[Title/Abstract] OR "serious mental disorder*"[Title/Abstract]) |
| CINAHL | (TX "Social isolat*" OR TX "Social exclu*" OR TX "Lonel*") AND (TX "Bipolar and Related Disorders" OR TX "Major depressive Disorder" OR TX "Schizophrenia Spectrum and Other Psychotic Disorders" OR TX "severe mental disorder*" OR TX "serious mental illness" OR TX "severe mental illness" OR TX "serious mental disorder*") |
| PsycInfo | (exp "social isolation"/ OR "social exclu*".ti,ab. OR "social isolat*".ti,ab. OR lonel*.ti,ab.) AND (exp "bipolar and related disorders"/ OR (exp "depressive disorder, major") OR exp "schizophrenia spectrum and other psychotic disorders"/ OR "severe mental disorder*".ti,ab. OR "serious mental illness".ti,ab. OR "severe mental illness".ti,ab. OR "serious mental disorder*".ti,ab.) |
| Web of Science | 1: TS=("Social exclu*")  2: TS=("Social isolat*")  3: TS=("Lonel*")  4: #1 OR #2 OR #3  5: TS=("Bipolar and Related Disorders")  6: TS=("Major depressive Disorder")  7: TS=("Schizophrenia Spectrum and Other Psychotic Disorders")  8: TS=("severe mental disorder*")  9: TS=("serious mental illness*")  10: TS=("severe mental illness*")  11: TS=("serious mental disorder*")  12: #5 OR #6 OR #7 OR #8 OR #9 OR #10 OR #11 OR #12  13: #4 AND #13 |
| Scopus | (TITLE-ABS-KEY("social exclu*") OR TITLE-ABS-KEY("social isolat*") OR TITLE-ABS-KEY("lonel*")) AND (TITLE-ABS-KEY("bipolar and related disorders") OR TITLE-ABS-KEY("major depressive disorder") OR TITLE-ABS-KEY("schizophrenia spectrum and other psychotic disorders") OR TITLE-ABS-KEY("severe mental disorder*") OR TITLE-ABS-KEY("serious mental illness") OR TITLE-ABS-KEY("severe mental illness") OR TITLE-ABS-KEY("serious mental disorder*")) |

# Supplement 2. Flowchart

**Identification of studies via databases and registers**

Records removed before screening:

Duplicate records removed (n = 890)

Records identified from*:

Databases (n = 4,506)

(Pubmed, n = 2,657)

(PsycINFO, n = 212)

(CINAHL, n = 212)

(Web of Science = 689)

(Scopus = 736)

**Identification**

Records screened

(n = 3,616)

Records excluded**

(n = 3,574)

Reports sought for retrieval

(n = 42)

Reports not retrieved

(n = 0)

**Screening**

Reports excluded: n=32

Prevalence not reported for loneliness/social isolation among individuals with severe mental disorder (n=30)

No peer-reviewed article (e.g., dissertation) (n=1)

Qualitative study (n=1)

Reports assessed for eligibility

(n = 10)

Studies included in review

(n =10, thereof n=1 included via hand search)

All n=10 studies were included in meta-analysis

**Included**

Reference: (Page et al. 2021)

# Supplement 3. Additional details regarding the included studies

| **Study** | **Assessment of loneliness/social isolation** | **Assessment of SMD** | **Sample and study Type** | **Results: antecedents/consequences** |
| --- | --- | --- | --- | --- |
| 1. Badcock et al., 2015 | Loneliness:  (“In the last 12 months have you felt lonely?” 4 response categories: “I have plenty of friends and have not been lonely”; “Although I have friends I have been lonely occasionally”; “I have some friends but have been lonely for company”; “I have felt socially isolated and lonely”. Only the first option was considered as “not lonely” in this study. | Psychotic disorders: In accordance with the ICD-10 criteria  (Schizophrenia, schizoaffective disorder, bipolar disorder with psychotic features, depressive psychosis and delusional disorder) | Cross-sectional survey (18 to 64 years) | Logistic regression showed that loneliness was associated with loss of pleasure (OR=2.0, 95% CI: 1.2 to 3.3, p<.05) and subjective thought disorder (OR: 1.4, 95% CI: 1.0 to 2.0, p<.05) |
| 1. Dell et al., 2019 | Loneliness: DJG tool (11-item version, 0 to 11; higher scores correspond to higher loneliness; scores of 2 or higher indicate loneliness | SMD: Diagnosed with SMD (mainly major depressive disorder, bipolar, schizophrenia) | Individuals receiving Community Psychiatric Rehabilitation Program services at a Community mental health center (Midwestern United States city; community-dwelling individuals aged 50 years and over fluent in English; not diagnosed with neurocognitive disorders)  Cross-sectional survey | Linear regressions showed that emotional loneliness was significantly associated with depressive symptoms (β=.74, p<.001), whereas social loneliness was not significantly associated with depressive symptoms (β=.40, p>.05). |
| 1. Fortuna et al., 2024 | Loneliness: UCLA-3 (3 to 9; 6 or higher were indicative for loneliness) | SMD: Primary ICD diagnosis data (F2x and F3x) were used based on electronic health records; Primary diagnoses were: Schizophrenia spectrum disorder, bipolar disorder and major depressive disorder | New York City boroughs (the Bridge, supporting housing agency: Manhattan, Bronx, and Brooklyn; community-dwelling; residents aged 50+ years)  Cross-sectional survey | Logistic regressions showed that age, gender, race, SMD diagnosis, and housing type were not significantly associated with high loneliness among the total sample.  However, logistic regressions showed that Hispanics (compared to White, OR: .079, 95% CI: .006 to .971, p=.047) and individuals residing in scattered site housing (compared to congregate, OR: 2.307, 95% CI: 1.131 to 4.707, p=.022) are associated with high loneliness among females. No significant associations with loneliness were present among men. |
| 1. Hameideh, 2021 | Loneliness: Revised UCLA Loneliness Scale (version 3, 20 items; total score ranges from 20 to 80). 20 to 34: Low level; 35 to 49: moderate level; 50 to 64: moderate to high level; 65 to 80: high level | Additional details not needed | Data from National Center for Mental Health (largest specialized psychiatric hospital in Jordan)  Cross-sectional survey (convenience sample)  In-patients | Linear regressions showed that higher loneliness was significantly associated with lower satisfaction with life (β=-.40, p<.001), low social support from friends (β=-.36, p<.001), higher duration of treatment (β=.15, p<.001). |
| 1. Heron et al., 2022 | Loneliness: UCLA-3 (from 3 to 9, 7 or higher were indicative for loneliness) and Single-Item (“How often do you feel lonely”, with options: “hardly ever”, “some of the time” and “often”) | Additional details not needed | Subsection of the “Closing the Gap (CtG)” clinical cohort: Optimising Well-being in Self-Isolation study (OWLS); recruited from 17 mental health trusts as well as six Clinical Research Networks (rural/urban settings in England)  Cross-sectional survey | Logistic regressions showed that loneliness was associated with living alone (compared to not living alone, adjusted OR = 2.04, 95% CI 1.21–3.43, p = .01), living in an area with high index of multiple deprivation (compared to an area with very low index, adjusted OR = 2.49, 95% CI 1.04–5.95, p = .04), and younger age (adjusted OR = -.98, 95% CI .964-.997, p = .02). |
| 1. Machetanz et al., 2023 | Objective social isolation: at least three of the following conditions need to be met (before admission): small social network; infrequent social contacts; absence of confidante connections; living alone; lack of participation in social activities | Additional details not needed | Court-mandated inpatient treatment (1982 to 2016, mostly from year 200 onwards) at the Centre for Inpatient Forensic Therapies of the University Hospital of Psychiatry Zurich  Retrospective design | A supervised machine learning model showed that attention disorder, alogia, crime motivated by ego disturbances, PANSS (positive and negative syndrome scale) score, and a history of negative symptoms can differentiate between patients with and without objective social isolation (balanced accuracy: 69%, AUC of .74). |
| 1. Okruszek et al., 2023 | Objective social isolation: LSNS-6 (0 to 30; fewer than 12 indicate objective social isolation) | Additional details not needed | A total of 93 adult patients with schizophrenia were enrolled in the study (comprising 29 inpatients from the Institute of Psychiatry and Neurology in Warsaw and 64 outpatients from the Outpatient Mental Health Clinic at the same institute  Cross-sectional survey  Cross-sectional survey | Structural equation model showed that social threat bias was associated with increased loneliness (UCLA-20) directly and indirectly via decreased social connection. |
| 1. Stain et al., 2012 | Additional details not needed | Additional details not needed | Cross-sectional survey (18 to 64 years) | Not reported |
| 1. Suman et al., 2023 | Loneliness: UCLA-3 (each rated on a 4-point scale; replying with (at least) “sometimes/often” (2) to any of the items reflects loneliness |  | Patients of schizophrenia in clinical remission (based on a patient group using outpatient services of a tertiary care facility; further inclusion criteria: in clinical remission, aged 18 to 65 years, ability to read Hindi/English)  Cross-sectional survey | Stepwise linear regressions showed that loneliness was largely explained by quality of life (29%), followed by hopelessness (8%) and discrimination (2%). |
| 1. Valeri et al., 2023 | Loneliness: Single-item (four response options from “not at all” to “extremely”). Highest two options indicate (moderate or extreme) loneliness. | SMD: Psychosis (Schizophrenia, Schizoaffective Disorder, Bipolar Disorder with Psychotic Features) | Adults with psychosis  (Intensive) longitudinal study | Logistic regressions showed that shelter-in-place orders were not significantly associated with increases in loneliness (OR=2.10, p=.08) |

Legend: AUC: Area Under the Curve; CI: Confidence Interval; DJG-tool: De Jong Gierveld loneliness tool; DSM: Diagnostic and Statistical Manual of Mental Disorders; ICD-10: 10^th^ Revision of the International Classification of Diseases; LSNS-6: Lubben Social Network Scale (6-item version); OR: Odds Ratio; PANSS: Positive and Negative Syndrome Scale; SMD: Severe Mental Disorder; UCLA-3: University of California, Los Angeles Loneliness Scale (3-item version); UCLA-20: University of California, Los Angeles Loneliness Scale (20-item version).

Included studies: (Badcock et al. 2015; Dell et al. 2019; Fortuna et al. 2024; Hamaideh 2021; Heron et al. 2022; Machetanz et al. 2023; Okruszek et al. 2023; Stain et al. 2012; Suman et al. 2023; Valeri et al. 2023)

# Supplement 4. Quality assessment/risk of bias assessment.

|  | Items | | | | | | | | | Quality score (from 0 to 9; higher scores indicate less risk of bias) |
| --- | --- | --- | --- | --- | --- | --- | --- | --- | --- | --- |
| Study | 1 | 2 | 3 | 4 | 5 | 6 | 7 | 8 | 9 | Total |
| 1. Badcock et al., 2015 | Y | Y | Y | Y | Y | Y | Y | Y | U | 8 |
| 1. Dell et al., 2019 | N | N | N | Y | Y | Y | Y | Y | Y | 6 |
| 1. Fortuna et al., 2024 | N | Y | Y | Y | Y | Y | Y | Y | U | 7 |
| 1. Hameideh, 2021 | N | N | Y | Y | Y | Y | Y | Y | U | 6 |
| 1. Heron et al., 2022 | Y | Y | Y | Y | Y | Y | Y | Y | U | 8 |
| 1. Machetanz et al., 2023 | N | N | Y | Y | Y | Y | Y | Y | U | 6 |
| 1. Okruszek et al., 2023 | N | N | N | Y | Y | Y | Y | Y | U | 5 |
| 1. Stain et al., 2012 | Y | Y | Y | Y | Y | Y | Y | Y | U | 8 |
| 1. Suman et al., 2023 | N | N | N | Y | Y | Y | Y | Y | U | 5 |
| 1. Valeri et al., 2023 | N | N | N | Y | Y | Y | Y | Y | U | 5 |

Legend: The Joanna Briggs Institute (JBI) standardized critical appraisal tool for prevalence studies was used. The criteria comprised the following aspects, each receiving a response of yes (Y), no (N), or unclear (U): 1 = Suitability of the sample frame; 2 = Suitability of participant selection; 3 = Sufficiency of sample size; 4 = Characterization of study participants and setting; 5 = Data analysis: appropriate coverage of the identified sample; 6 = Employment of valid methods for condition identification; 7 = Standard/reliable evaluation of the condition; 8 = Suitability of the analytical method; 9 = Sufficiency of the response rate or appropriate handling of a low response rate, if needed.

Included studies: (Badcock et al. 2015; Dell et al. 2019; Fortuna et al. 2024; Hamaideh 2021; Heron et al. 2022; Machetanz et al. 2023; Okruszek et al. 2023; Stain et al. 2012; Suman et al. 2023; Valeri et al. 2023)

# References

**Badcock JC, Shah S, Mackinnon A, Stain HJ, Galletly C, Jablensky A and Morgan VA** (2015) Loneliness in psychotic disorders and its association with cognitive function and symptom profile. *Schizophrenia research* **169**(1-3)**,** 268-273.

**Dell NA, Pelham M and Murphy AM** (2019) Loneliness and depressive symptoms in middle aged and older adults experiencing serious mental illness. *Psychiatric rehabilitation journal* **42**(2)**,** 113.

**Fortuna KL, Rhee TG, Leininger LJ, Ferron J, Elwyn G, Raue PJ, Heller R and Werlin J** (2024) Estimates of loneliness among racially and ethnically diverse adults with serious mental illness in New York City boroughs: Manhattan, Bronx, and Brooklyn. *Journal of the American Geriatrics Society* **72**(3)**,** 924-927.

**Hamaideh SH** (2021) Loneliness Among in-patients Diagnosed With Schizophrenia: Its Correlates and Relations With Social Support and Satisfaction With Life. *Malaysian Journal of Medicine & Health Sciences* **17**(1).

**Heron P, Spanakis P, Crosland S, Johnston G, Newbronner E, Wadman R, Walker L, Gilbody S and Peckham E** (2022) Loneliness among people with severe mental illness during the COVID-19 pandemic: Results from a linked UK population cohort study. *PLoS One* **17**(1)**,** e0262363.

**Machetanz L, Lau S, Huber D and Kirchebner J** (2023) Correlates of Social Isolation in Forensic Psychiatric Patients with Schizophrenia Spectrum Disorders: An Explorative Analysis Using Machine Learning. *International Journal of Environmental Research and Public Health* **20**(5)**,** 4392.

**Okruszek Ł, Piejka A, Chrustowicz M, Krawczyk M, Jarkiewicz M, Schudy A, Ludwig K and Pinkham A** (2023) Social cognitive bias increases loneliness both directly and by decreasing social connection in patients with schizophrenia. *Schizophrenia research* **256,** 72-78.

**Page MJ, McKenzie JE, Bossuyt PM, Boutron I, Hoffmann TC, Mulrow CD, Shamseer L, Tetzlaff JM, Akl EA, Brennan SE, Chou R, Glanville J, Grimshaw JM, Hróbjartsson A, Lalu MM, Li T, Loder EW, Mayo-Wilson E, McDonald S, McGuinness LA, Stewart LA, Thomas J, Tricco AC, Welch VA, Whiting P and Moher D** (2021) The PRISMA 2020 statement: an updated guideline for reporting systematic reviews. *Bmj* **372,** n71.

**Stain HJ, Galletly CA, Clark S, Wilson J, Killen EA, Anthes L, Campbell LE, Hanlon M-C and Harvey C** (2012) Understanding the social costs of psychosis: the experience of adults affected by psychosis identified within the second Australian National Survey of Psychosis. *Australian & New Zealand Journal of Psychiatry* **46**(9)**,** 879-889.

**Suman A, Nehra R, Sahoo S and Grover S** (2023) Prevalence of loneliness and its correlates among patients with schizophrenia. *International Journal of Social Psychiatry* **69**(4)**,** 906-915.

**Valeri L, Rahimi-Eichi H, Liebenthal E, Rauch SL, Schutt RK, Öngür D, Dixon LB, Onnela J-P and Baker JT** (2023) Intensive longitudinal assessment of mobility, social activity and loneliness in individuals with severe mental illness during COVID-19. *Schizophrenia* **9**(1)**,** 62.
